# Pelvic Shadowing as a Diagnostic Predictor of Orthopedic Pathology in Orthopedic Trauma Patients

**DOI:** 10.7759/cureus.17873

**Published:** 2021-09-10

**Authors:** Brian Skura, Michael P Ebaugh, Braden J Passias, Daniel DeGenova, Adam Hoffman, Joseph Scheschuk, Benjamin C Taylor

**Affiliations:** 1 Orthopedic Surgery, OhioHealth, Columbus, USA; 2 Foot and Ankle Reconstruction/Orthopedic Surgery, Orlando Health Jewett Orthopedic Institute, Orlando, USA; 3 Orthopedic Surgery, OhioHealth Grant Medical Center, Columbus, USA; 4 Orthopedic Trauma, OhioHealth Grant Medical Center, Columbus, USA

**Keywords:** orthopedic trauma, radiology, lower extremity trauma, pelvic shadowing, throckmorton

## Abstract

Introduction

The Throckmorton sign, or John Thomas sign, is a well-established orthopedic eponym, anecdotally used in orthopedic surgery to correlate the direction of male genitalia, observed on a pelvic radiograph, with the laterality of an associated orthopedic pathology. In earlier studies, the direction of pelvic shadowing on X-ray has been neither a credible nor a reliable predictor of fracture laterality. Given this small body of evidence, we sought to further investigate the relationship between peri-trochanteric hip fracture laterality and male genitalia lie.

Method

A single-center retrospective chart review was conducted of 397 consecutive male patients who received pelvic radiographs performed upon entry to an urban level 1 trauma center. Exclusion criteria included age less than 18 years or a prior history of pelvic or urological surgery. Of this cohort, 360 patients met the inclusion criteria and underwent investigation.

Results

The study population had an average age of 42 years (range: 18-91 years). Statistical analysis yielded a 4.24 relative risk with pelvic shadowing laterality and respective peri-trochanteric hip fracture sidedness. Additionally, there was a 4.63 and 9.88 relative risk of tibial shaft fractures and distal radius fractures having a concomitant positive Throckmorton Sign, respectively.

Conclusion

Pelvic shadowing can be used as an additional diagnostic tool in predicting peri-trochanteric hip fracture sidedness in a trauma bay setting.

## Introduction

In the United States, trauma is the leading cause of death in individuals under the age of 45 years [1]. The overwhelming majority of multiply injured trauma patients are middle-aged males, sustaining craniocerebral (60.7%) and thoracic (61.9%) trauma [2]. These injuries often result in the decline of neurologic and respiratory status, making it difficult for the patient to adequately communicate the location of pain and thus increasing the risk of missed orthopedic injuries. Accurate diagnosis of fractures can be exhaustive in a non-communicative patient often requiring multiple diagnostic modalities.

Anteroposterior (AP) chest and pelvic radiographs are a mainstay of the secondary survey conducted per the Advanced Trauma Life Support (ATLS) treatment algorithm. Findings on these radiographs may lead to further studies and focused examinations [3]. A great deal of information can be gained from primary radiographs obtained in trauma patients. Although subtle at times, soft tissue changes can be a valuable marker of occult injury. In males, the laterality of pelvic shadowing seen on AP pelvis radiographs has been previously postulated as a possible predictor of hip fracture laterality [4].

There have been studies attempting to determine the accuracy of using the pelvic shadow and hip fracture sidedness; however, there have not been any studies of this sample size looking at pelvic shadowing as being predictive of not only hip fractures but also complete appendicular and axial skeletal trauma. We hypothesize that the position of pelvic shadow could be used as a reliable predictor of peri-trochanteric fracture laterality, without correlation with more distal appendicular trauma.

## Materials and methods

A retrospective chart review was conducted of 360 male patients who underwent radiographic pelvic evaluation and subsequent orthopedic stabilization at an urban level 1 trauma center. Prior to initiation of this study, the study was determined to be exempt by the Institutional Review Board. No outside funding was received for this study. Informed consent was waived by OhioHealth Research Institute. All research was performed in accordance with all regulatory guidelines and regulations.

All patients were evaluated for a traumatic orthopedic injury between December 1, 2016, and August 31, 2017, who also had an initial AP pelvic radiograph at the time of the ATLS primary survey.

**Figure 1:**
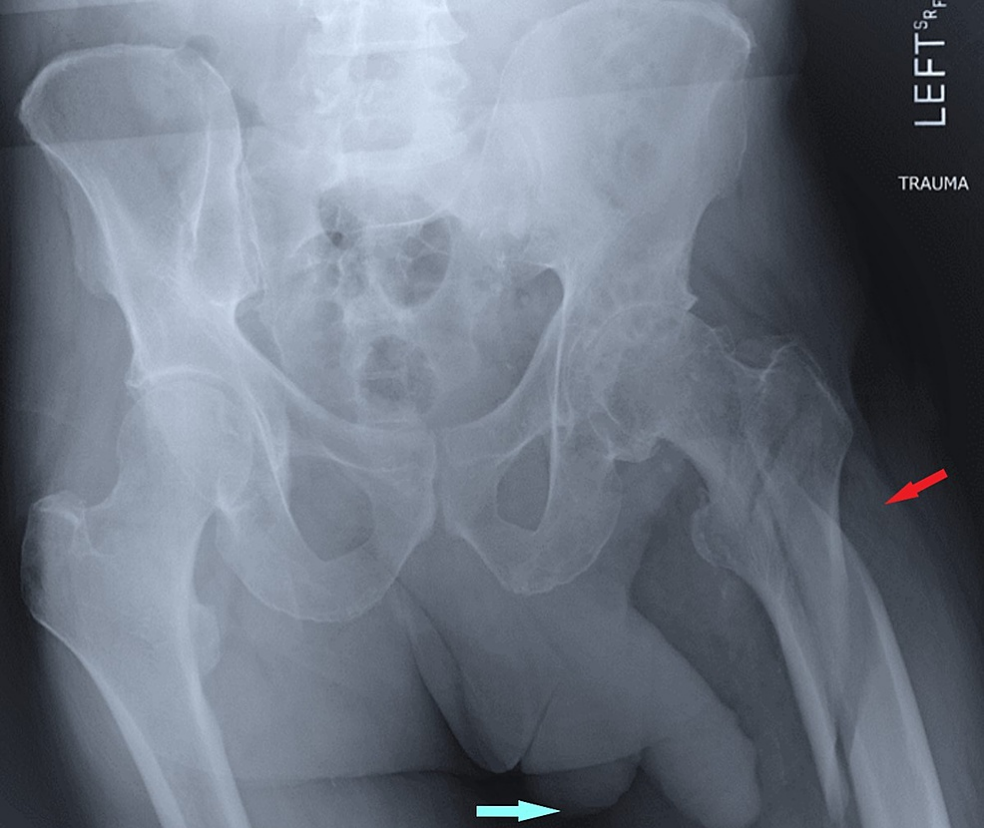
Anteroposterior radiograph of the pelvis demonstrating left-sided pelvic shadowing to the ipsilateral peri-trochanteric hip fracture. The red arrow is pointing to the fracture. The blue arrow is pointing in the direction of the pelvic shadowing towards the side of the fracture.

Patients aged ≤18 years or with a prior history of pelvic or urological surgery were excluded from the investigation. Electronic and written medical records were utilized to collect patient data, which was assembled in a database (Microsoft Excel, Redmond, WA). Patient demographics, fracture and injury characteristics, operative variables, radiographic information, and post-operative outcome measurements were recorded for each patient assessed in the study. A single investigator was selected to evaluate the "side of lie" in each AP pelvic radiograph. The investigator noted either right, left, or neither. An example of an AP pelvic radiograph with a left-sided penile lie is noted in Figure 1. It was hypothesized that pelvic shadowing would be a diagnostic marker for the presence of underlying injury, and its respective laterality, in male orthopedic trauma patients.

Statistical analysis was performed, with means, ranges, and confidence intervals calculated for continuous variables and compared using Student’s t-tests. Frequencies were calculated for continuous variables and compared using Fisher’s exact test for increased accuracy in small proportion analysis. A significance level of ≤0.05 was set as significant.

## Results

The average age of this investigative cohort was 42 years (range: 18-91 years), with a total of 360 males who presented to this level 1 trauma center. The most common mechanism of injury was found to be motor vehicle collision, and lower extremity trauma was the most affected (area or injury) in these individuals. Demographic data and injury characteristics are given in Table 1.

**Table 1:** Demographic and injury data Values are expressed as means with ranges in parentheses or as absolute values with percentages in parentheses.

Characteristics	
Age (years)	42.2 ± 16.8 (18-91)
Sex (male)	360 (100%)
Glasgow Coma scale	14.1 ± 2.7 (3-15)
Penetrating injury	21 (5.8%)
Mechanism of injury	
Motor vehicle collision	156 (43.3%)
Motorcycle collision	51 (14.2%)
Fall	65 (18.1%)
Assault	8 (2.2%)
Crush	15 (4.2%)
Bicycle	5 (1.4%)
Gunshot	18 (5%)
Pedestrian versus car	22 (6.1%)
Other	20 (5.6%)
Injury pattern	
Upper extremity only	107 (29.7%)
Lower extremity only	171 (47.5%)
Multiple extremities	82 (22.8%)
Rib fractures	55 (15.3%)
Pelvic ring or acetabular fracture	48 (13.3%)

The following three fracture patterns that demonstrated significant association with the side of pelvic shadow lay and fracture sidedness: peri-trochanteric hip fractures (RR = 4.24; p = 0.04), tibial shaft fractures (RR = 4.63; p = 0.04), and distal radius fractures (RR = 9.88; p = 0.01). Pelvic ring injuries and acetabular fractures demonstrated an association with pelvic shadow sidedness (RR = 7.01 and 4.28, respectively); however, it was not statistically significant. No other specific fracture patterns captured in this study were found to have a significant association with the side of pelvic shadow lay and fracture sidedness. All fracture patterns and associated relevant statistical analysis can be seen in Table 2.

**Table 2:** Pelvic shadowing laterality and associated direction of orthopedic fracture sidedness Values are expressed as p-values with significant level set to p < 0.05

Type of injury	Relative risk
Penetrating injury	0.05
Axial skeleton injuries	
Pelvic ring injury	0.13
Rib fractures	0.71
Acetabular fractures	0.33
Sternal fractures	0.1
Upper extremity injuries	
Distal radius fractures	<0.01
Forearm fractures	0.29
Elbow fractures	0.23
Humerus fractures	0.7
Scapula fractures	0.22
Clavicle fractures	0.18
Lower extremity injuries	
Midfoot fractures	0.27
Calcaneus fractures	0.73
Talus fractures	1
Rotational ankle fractures	1
Pilon fractures	0.35
Diaphyseal tibia fractures	0.04
Tibial plateau fractures	0.58
Knee arthrotomy	1
Distal femur fractures	0.5
Diaphyseal femur fractures	0.24
Peri-trochanteric fractures	0.04
Femoral neck fractures	0.21

Penetrating injuries were 2.6 times more likely to be associated with a right-sided pelvic shadow (p = 0.03). Right-sided pelvic shadow was found to be 2.61 times more likely in all left upper extremity injuries, but there was no significant association with specific fracture patterns. No significant association was found between same-side pelvic shadow and ipsilateral extremity injury.

## Discussion

Pelvic shadowing is synonymous with the well-known “Throckmorton” or “John Thomas” sign. Typically, within the medical community, signs, results, and techniques that have been touted as unreliable or insignificant have been used as examples of things to avoid and cast off to be forgotten. The “Throckmorton” and “John Thomas” signs have stood the test of time and secured their spot in popular medical culture.

Pelvic shadowing was first hypothesized to play a role as a soft tissue marker for hip fracture sidedness based on penile position, that is, the fractured hip would lead to a shortened, externally rotated leg and thus tilt the pelvis to the affected side and bring the lay of the penis to that side [4]. The positional hypothesis did not take into consideration multiply injured patients, urogenital injury, or the mechanism of injury. This investigation set out to determine whether pelvic shadowing can be used as a reliable predictor in the trauma setting for hip fracture sidedness as well as appendicular and axial skeletal fractures.

Studies have shown that 2-10% of proximal femur fractures may be missed on initial radiographs, especially in the trauma setting [5,6]. Unlike other joints, where the presence of an effusion on plain radiograph of the ankle [7] or a displacement of the posterior fat pad in the elbow is informative [8,9], there has yet to be a reliable radiographic sign at the hip to indicate an occult hip fracture. Occult proximal femur fractures can lead to devastating consequences, complicating surgical intervention and comprising ideal outcomes [10]. Unfortunately, there is little substantial evidence within the literature regarding the use of pelvic shadowing as a reliable soft tissue change to predict laterality of hip pathology or injury. Several studies have failed to demonstrate a correlation between pelvic shadowing and hip fracture laterality, with one study describing this sign as “less accurate than the toss of a coin” [11-15]. Murphy et al. attempted to find a relationship between pelvic shadowing and not only with hip fracture side but also penile size and angulation. They retrospectively reviewed AP pelvis radiographs of 200 males, of which 100 had a hip fracture and 100 were controls. Their results showed only an accuracy of 46%, and their conclusions determined that this sign is no better than the toss of a coin in relation to hip fractures [11]. These findings are similar to a study conducted by Ya'ish and Baloch who retrospectively reviewed pelvic radiographs of 100 males with hip fractures and compared them with 100 males without hip fractures, and noted the direction of penile shadowing with a sensitivity of only 30% [13]. Brink et al. conducted a retrospective study where four male physicians and one female physician were blinded to reading the AP X-rays of the pelvis of 51 male patients with hip and pelvic disorders and compared them to 11 X-rays without pathology. They found the sensitivity of all reviewers to be 55% for hip pathology and concluded that using this sign was not useful in the diagnosis of hip fractures [12]. On the contrary, there have been some studies with opposing results that could be deemed outliers.

Sooloki and Vosoughi reviewed 500 radiographs of men with single fractures in the lower limb. They found this sign to be positive in 87.8% of patients with hip fractures and also noted tibial fractures to have a positive sign in 90% of patients analyzed in the same data sets [15]. Similarly, Mouzopoulos found this sign to be positive in 96.2% of patients with a displaced intertrochanteric (IT) fracture and 93.2% in displaced subcapital fractures, and concluded that this sign is attributed to the turn of the pelvis on the fractured side [14]. Although this study found a high correlation between pelvic shadowing sidedness and displaced peri-trochanteric fractures, this was done in low-energy, elderly hip fractures and did not include any patient below the age of 68 years.

We found a significant association between same-side pelvic shadow lay and peri-trochanteric fractures (including both IT and subtrochanteric fractures) in a population with a mean age of 42 years. The findings demonstrated that same-side lay occurred 4.24 times more often than the lay being found on the contralateral side of the fracture. This study design expanded on the principal hypothesis that pelvic shadowing was only a predictor of hip fracture sidedness by examining the relationship of pelvic shadowing with other parts of the body. We found pelvic shadowing to also be a predictor of distal radius and diaphyseal tibia fractures (RR= 9.88, p = 0.01 and RR = 4.63, p = 0.04, respectively). Interestingly, there was an association between right-sided lay and left upper extremity injury (RR = 2.61). Unfortunately, as groups, ipsilateral upper extremity and lower extremity fracture sidedness were not found to be predicted by pelvic shadowing.

To our knowledge, this is the largest study to date analyzing pelvic shadowing as a potential diagnostic tool in a patient population with a younger average age and more diverse mechanism of injury compared with other studies at large level 1 trauma centers.

Due to the larger sample size, the findings were strongly powered and showed significant results; however, there are several limitations to this investigation. First, this study is subject to the inherent limitations tied to its retrospective nature. In addition to having a single investigator evaluate the “side of lie,” certain situations can obscure the interpretation of soft tissue orientation with respect to other orthopedic injuries. Although infrequent, body habitus, overlying clothing, and pelvic binders could provide difficulty with accurate radiographic interpretation. Within the community setting, a patient is unlikely to receive an AP radiograph of the pelvis for an upper extremity injury, such as a distal radius fracture. Therefore, this sign is unlikely to aid in the final diagnosis of an obvious extremity fracture in a patient who can clearly express the location of their pain.

## Conclusions

This study demonstrated that pelvic shadowing can be used as an additional diagnostic tool in determining peri-trochanteric fracture sidedness in the trauma setting, particularly in a non-communicative patient. Expeditious and accurate decision-making is crucial when dealing with the multiply injured patient in the trauma setting. Using every available resource to aid in accurate diagnosis of orthopedic injuries can mean the difference between a good outcome and devastating complications. Further prospective investigations are needed to elucidate the cause of the side of lie.
